# Perceptions of Dietary Habits and Risk for Type 2 Diabetes among Congolese Immigrants

**DOI:** 10.1155/2017/4736176

**Published:** 2017-11-12

**Authors:** Daudet Ilunga Tshiswaka, Kelechi D. Ibe-Lamberts, Dyna Miandabu Mulunda, Juliet Iwelunmor

**Affiliations:** ^1^Department of Public Health, University of West Florida, 11000 University Parkway, Pensacola, FL 32514, USA; ^2^Department of Psychology, University of Florida, 114 Psychology Building, P.O. Box 112250, Gainesville, FL 32611-2250, USA; ^3^Department of Kinesiology and Community Health, University of Illinois at Urbana-Champaign, 110 Huff Hall, 1206 South Fourth St., Champaign, IL 61820, USA; ^4^Department of Behavioral Science and Health Education, Saint Louis University, Salus Center, 3545 Lafayette Ave., St. Louis, MO 63103, USA

## Abstract

**Objective:**

To explore the perceptions of dietary habits and type 2 diabetes risk among Congolese immigrants living in the US.

**Methods:**

Data were collected from 20 in-depth interviews and photo-elicitation techniques conducted with Congolese immigrants. The PEN-3 cultural model was used as a guide to analyze the data collected.

**Results:**

Participants identified positive, existential, and negative perceptions, enablers, and nurturers associated with dietary habits and type 2 diabetes risk. Participants also acknowledged intrinsic cultural ways of understanding and interpreting the interaction between dietary habits and type 2 diabetes risk among the Congolese people which may influence their health-seeking practices.

**Conclusions:**

The findings underscore the importance of culture and how sociocultural factors may play a role with designing culturally appropriate interventions aimed at addressing the risk for type 2 diabetes among Congolese immigrants in the US.

## 1. Introduction

Presently, the incidence of type 2 diabetes is increasing at an alarming rate within the United States (US) and also on a global scale [1, 2]. According to the International Diabetes Federation [3], the global population of adults with both type 1 and type 2 diabetes is projected to increase from 382 million in 2013 to 592 million by 2035, with type 2 diabetes specifically accounting for 90–95% of cases [4]. Immigrants worldwide are experiencing this increased risk for type 2 diabetes due to lifestyle changes [5]. According to the CDC [6], small cities in the US like Champaign, Illinois—which has a relatively large number of Congolese immigrants [7, 8]—had a proportion of diagnosed diabetes that has risen slightly from an estimated 7.1% in 2009 to 8% in 2013. While diabetes in Champaign is more prevalent among minorities [9], the prevalence of diabetes among immigrants from the Democratic Republic of Congo (DRC) living in the Champaign area remains unstudied. Yet, the DRC reported a prevalence of 1.7 million cases of diabetes out of a total 14 million diabetics in Africa in 2015. That is, the prevalence of diabetic adults was estimated at 5.3% in DRC [10]. Despite relatively high diabetes proportion reported in the country of origin, Venters and Gany [11] discovered that the prevalence of diabetes among African immigrants is not widely reported in the country of resettlement. Additionally, the prevalence of overweight and obesity diagnoses—often a consequence of losing traditional dietary habits and adapting unhealthy behaviors— is found to be a contributing factor to the epidemic.

Commonly, immigrants tend to travel to developed countries where epidemiological transition, increased urbanization, and other environmental factors can often influence rapid changes in lifestyle, including dietary habits [12]. Choukem et al. [5] argued that swift changes in lifestyle, along with a genetic background, can increase the likelihood of developing chronic diseases such as type 2 diabetes. Additionally, the development of type 2 diabetes in immigrants is tied to metabolic alterations resulting from changes in dietary habits—the habitual decisions of individuals or groups of people regarding what foods they eat [13]. With dietary habits changing drastically due to their emigration, immigrants such as the Congolese in the US are at great risk of developing type 2 diabetes compared to those who remained in their country of origin [5].

For African immigrants, an additional factor influencing the increased incidence of type 2 diabetes is culture. In some sub-Saharan African culture, being overweight or obese is sometimes considered a sign of wealth [14, 15]. The interaction between body weight and wealth is a concept that serves as an example of the existential cultural perceptions present in communities like the Congolese immigrant group. Due to this cultural belief, host countries could face challenges implementing diabetes prevention programs because of potential conflict with cultural perceptions regarding health and body weight among African immigrants [16]. The intersection of (a) post-resettlement changes in dietary habits and (b) engaging in a more sedentary lifestyle makes designing culturally appropriate interventions necessary and challenging [11].

The purpose of this study was to explore the perceptions of dietary habits and type 2 diabetes risk among Congolese immigrants living in the US. We believe that understanding how cultural perceptions affect dietary behaviors of Congolese would be foundational and a stepping stone for future exploration on topics in this population such as body image and exercise. We used the PEN-3 cultural model as a guide to examine the sociocultural factors associated with Congolese immigrants' perceptions of diet and type 2 diabetes risk.

## 2. Materials and Methods

### 2.1. Conceptual Framework

The PEN-3 model was the conceptual framework used to guide the analysis of the current investigation. This model has been successfully used in studies that underscored the importance of culture in health [17]. Thus, the model is primarily used as a framework for health promotion and disease prevention strategies that emphasize the role of culture. The PEN-3 model has three domains: *cultural identity*, *relationships and expectations*, and *cultural empowerment*. The PEN-3 domains have proven to be specifically effective in highlighting the role and importance of cultural factors within perceptions of diabetes risk factors [18]. According to Airhihenbuwa [17], the *relationships and expectations* domain categorizes insights as *perceptions*, *enablers*, and *nurturers*, while the *cultural empowerment* domain assesses beliefs by classifying them as *positive*, *existential*, and *negative*. The *cultural identity* domain serves primarily as the intervention points of entry for developed strategies and can occur at the level of *persons*, *extended family members*, or *neighborhood* [17]. For the purpose of this study, the *relationships and expectations* and *cultural empowerment* domains were relied on for the needs assessment.

### 2.2. Study Design

This qualitative study utilized photo-elicitation supplemented with in-depth interviews to collect data from Congolese immigrants aged 35 years or older. The rationale behind these ages is justified by recommendations of the American Diabetes Association for people of African descent or other at-risk individuals for immediate type 2 diabetes screening [19]. Photo-elicitation is an interviewing technique that uses visual images (e.g., photos, videos, and paintings) to spark conversation among study participants and the interviewer [20]. Hatten et al. [21] proclaimed that pictures often capture experiences that shape the way people think. In this study, three researchers (the primary researcher and two independent researchers) selected pictures used for photo-elicitation and pilot-tested the images to ensure that the experiential focus of the investigation was captured.

This study was approved by the University of Illinois Institutional Review Board. Upon completion of informed consents from each participant, first photo-elicitation was used to spark a conversation about perceptions of risk factors for type 2 diabetes and followed by in-depth interview using questions guided by the PEN-3 model.

### 2.3. Participants

Twenty (20) Congolese immigrants participated in this study and were divided equally by gender (10 males and 10 females). Recruitment took place by telephone or word of mouth in Champaign county, Illinois, where relatively high concentrations of Congolese immigrants live [7]. Participants were prescreened as nondiabetics; the study objective was to derive perceptions of dietary habit around type 2 diabetes prior to any diagnoses of type 2 diabetes. Demographic data was collected for participants, including marital status, employment status, and residence status. It has been shown that these factors can correlate with the risk factors of diabetes [22].

### 2.4. In-Depth Interview Protocol

Photo-elicitation and in-depth interview sessions were conducted at times and locations convenient for participants. Each session lasted approximately 60 minutes. Two trained Congolese researchers conducted separate interviews and photo-elicitation sessions in French for men and women, respectively. Interviews were conducted in French because French is the preferred language of communication for most Congolese. Before the commencement of interviews, demographic data was collected. Interviews were (a) audio-recorded with permission from participants, (b) transcribed verbatim in French, and (c) translated in English by third party individuals to avoid translation bias by the researchers. There were 3 questions for the photo-elicitation process and 13 open-ended questions (including probes) for in-depth interviews (Table 1) which sought to capture participants' perceptions about the interaction between their dietary behaviors and risk for type 2 diabetes. Saturation was reached with the 20 persons interviewed through photo-elicitation and in-depth interview.

### 2.5. Data Analysis

Using NVivo 11 software for the qualitative analysis of data, three researchers triangulated the data by reading the English transcripts, coding them, highlighting conceptual labels, creating categories, and identifying emergent themes that evolved from the data. All independent researchers were in agreement with emergent themes to ensure inter-coder reliability. These themes were then situated within the conceptual framework in order to ground the findings in theory. Analyzed data were presented back to 10 participants (5 males and 5 females) for member checking, which is a crucial technique in establishing trustworthiness and validity in qualitative approach [23]. Participants reported that the interpretation of phenomenon and themes reflected in the analysis accurately resonated their perspectives.

## 3. Results

Table 2 summarizes the basic demographic characteristics of study participants. Overall, we found that the mean age was 43.7 ± 6.63 and 44.7 ± 5.46 years among male and female participants, respectively.

Only two domains (*relationships and expectations* and *cultural empowerment*) were used to analyze the qualitative data collected. The *cultural identity* domain*—*that refers to the intervention points of entry [18]—was not used. The emergent themes were categorized according to the PEN-3 domains (i.e., *relationships and expectations* and *cultural empowerment*). Each domain contains three subcategories. For instance, *relationships and expectations* has perceptions, enablers, and nurturers as subcategories, while *cultural empowerment* has positive, existential, and negative for subcategories.

### 3.1. Perceptions

**Figure 1:**
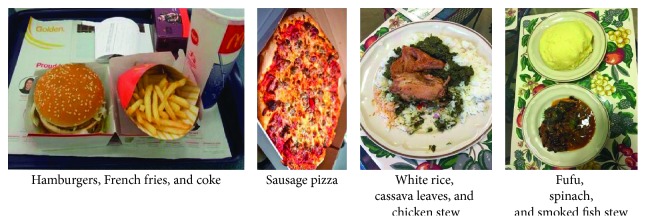


According to Airhihenbuwa and Webster [24], perceptions refer to knowledge, attitudes, and beliefs toward dietary habits and type 2 diabetes risks. In this study, participants commonly perceived American fast foods as a factor that can influence poor health and risks for type 2 diabetes (Figure 1). With the consumption of westernized foods in restaurant settings—despite concerns of the effect added ingredients have on their health outcome—our findings suggest that participants believed “westernized” or American foods should only be consumed occasionally to limit the risk for type 2 diabetes. One participant shared the following:

*I think this (pizza) is good food but not good to consume every day because I always fear with all the added ingredients, which cannot be good for my health…that's why I gave a detail I say no! No! I do not like pork, do not put pork on my pizza because I think pork will give me more fat.* (Male, 45 years)

Another participant stated that

*I think that everything they sell in the fast food restaurants, they do not think about the effect it can bring to consumers and as we prepare at home then I think that I can eat it (happy meal)…occasionally, so once a month, that's fine…because it will increase the risk for diabetes. *(Female, 46 years)

In addition, participants suggested that eating traditional Congolese foods was protective towards type 2 diabetes risks. Participants linked their Congolese identity to healthy food choices and minimal dietary acculturation (the process by which immigrants adopt the dietary practices of the host country) [25]. For example, participants suggested

*I am proud to be Congolese because we eat well, fish, vegetables that decrease the chance of developing diabetes. We eat the right things and I, personally, continue to eat the same thing. For us, we are not used to eat too much meat.* (Male, 42 years)

*I eat foods that I started eating since I was a child in DRC. I think it is good, it is better and if I continue to eat the same type of foods I wouldn't develop diabetes as there is not too much sugar in them.* (Female, 40 years)

*Yeah so the quality of the food in my case for example I eat much more African [Congolese] foods where there is... where it's natural food, where there is no excess calories. So I think it's good! …I wonder if it is not for this reason that I keep a bit the same size.* (Male, 58 years)

These comments stressed the belief around the relationship between type 2 diabetes and specific foods. For some participants, diabetes was described as the excessive consumption of sweetened food or food products. For instance, participants shared the following:

*I think diabetes is primarily the excess of sugar in the body. The word diabetes evokes sugar and where there is a high consumption of sugary foods.* (Male, 38 years)

*Yeah, because if we do not eat properly ... if we do not control diet and we have added sugar levels in the body and this is what causes diabetes.* (Female, 45 years)

*I know diabetes is caused by excessive consumption of sweetened foods like cakes, candy, and soda.* (Male, 58 years)

*For me, I try to stay away from things like cookies and sodas to avoid diabetes, I prefer to drink water.* (Female, 42 years)

### 3.2. Enablers

Enablers are defined as resources and institutional forces in the community that encourage or discourage dietary habits that can influence risk for type 2 diabetes [24]. In our study, participants acknowledged that they lived close to grocery stores; however, the majority of these stores did not provide healthy food options that could minimize risks for type 2 diabetes. Participants believed grocery stores, establishments in various communities, are mostly comprised of unhealthy, sweetened, food products and lack indigenous ingredients used to prepare healthier traditional food. For example, some participants shared the following:

*Grocery stores are not too far from here…Not me personally, I find that stores do not help people to eat well, because at the entrance there are always sweet stuffs.* (Female, 42 years)

*I don't have a problem accessing the grocery stores at all, but I have problem finding my Congolese foods in the stores, especially those big stores.* (Male, 40 years)

Another participant noted the following:

*Grocery stores are generally clean, but they have a lot of sweet stuff that […] expose us to diabetes.* (Male, 50 years)

Furthermore, participants described misinformation as a factor between their dietary habits and risk for type 2 diabetes. Participants mentioned the reason they relied on health care professionals or doctors for information is because they have been trained to understand the factors that affect diet and create type 2 diabetes risk. Our findings highlight that participants discussed resources in the community they rely on for health information. Some participants noted that they relied on health care professionals for correct information concerning risk for type 2 diabetes. For instance, participants stated

*I prefer to talk to specialists, a specialist doctor. Yes, because he studied this matter [diet and diabetes risk] and has the expertise. Rather than to go talk to someone, who can misguide me and can give me any ideas that are not verifiable and can be dangerous.* (Male, 58 years)

*I go to the hospital when I have questions about diabetes, I go to the doctor. Doctors can give us information about the disease. They can tell us here are the symptoms of the disease.* (Female, 35 years)

### 3.3. Nurturers

Nurturers refer to the role family members or friends play with supporting and/or discouraging dietary habits likely to influence type 2 diabetes risks [24]. The majority of participants mentioned that their parents influenced perceptions and values they have regarding their dietary habits and type 2 diabetes risk. Additionally, their parents emphasized the importance of limiting the consumption of food high in sugar and eating more “bitter” vegetables as a way of preventing type 2 diabetes. Participants also acknowledged first learning about type 2 diabetes risk and dietary habits from their parents. These views were illustrated with the following quotes:

*Even our parents told us when we were growing up to avoid consuming lots of sugar. Well, when we were young, our parents were saying that we should eat well, even in Kinshasa, we were asked to eat Bilolo [a bitter vegetable] is good as it prevents diabetes.* (Male, 42 years)

*[…] As always when we were children we were told by our parents if you take too much sugar you'll have diabetes.* (Female, 45 years)

*My mom used to tell me not to put too much sugar in my tea, as this will give me diabetes.* (Male, 35 years)

*When I was a child, my parents wanted me to eat bitter foods instead of consuming sweetened foods to avoid diabetes. *(Female, 38 years)

### 3.4. Positive

**Figure 2:**
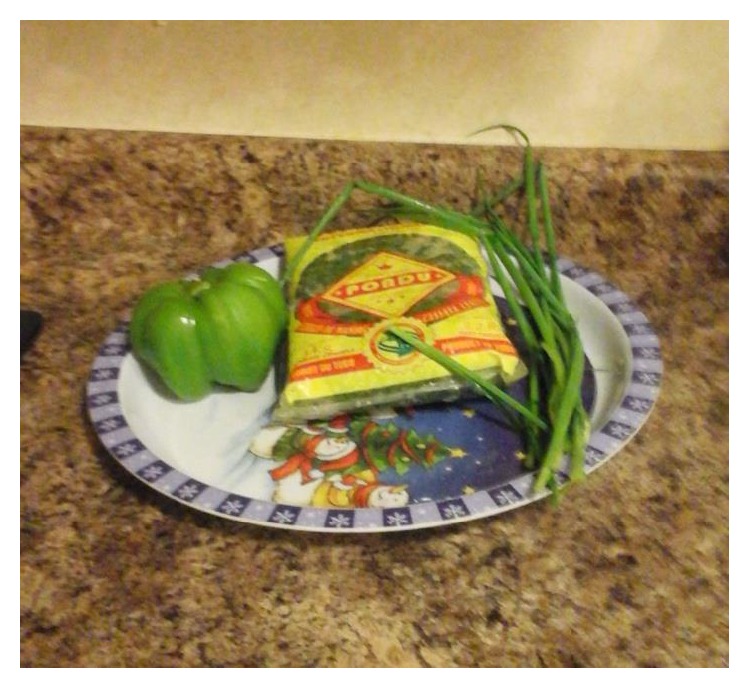


The positive dimension refers to values and relationships that promote healthy dietary habits and reduce type 2 diabetes risks [24]. Findings suggest that participants perceived Congolese dietary habits as healthy because its organic ingredients could positively influence health (Figure 2). Participants noted that consuming naturally grown food promotes healthy dietary habits that reduce type 2 diabetes risk. Participants shared the following statements:

*It [Congolese food] is organic, we eat produces grown naturally, without fertilizers and that's really the strong point of the Congolese foods. We're used to eating “domestic poultry” but here I don't know even what to say about chickens that grow within one week. These are the things that in the long run, we don't even know if that can cause disease.* (Female, 42 years)

*We eat produces that have been grown naturally with less fertilizers and chemicals. I think consuming organic produces protect us from developing diabetes.* (Male, 35 years)

Participants also mentioned the benefits of consuming specific vegetables in order to control blood sugar levels. For example, some tribes have traditional, indigenous vegetables that are believed to lower sugar levels. It was mentioned by some participants that the consumption of those vegetables serves as both controlling and protective factors for diabetic and nondiabetic individuals, respectively. One participant summarized this view by saying the following:

*Especially in Kinshasa there is Fumbwa [eru or Gnetum africanum, a local vegetable]. For example, I have the case of a brother-in-law who was in Italy he had diabetes. He came to Kinshasa and they advised him to consume Fumbwa; He took some, he found that it (Fumbwa) had reduced his sugar level... He went to Italy; he had his friend, who had two children who suffered of diabetes, when he came to Kinshasa and he bought a significant amount of Fumbwa to bring to Italy.* (Male, 47 years)

Another participant said this:

*In our tribe Yanzi [a tribe from the south-west part of the country], we have roots known as Mizinzi, a legume that helps lowering sugar level and not develop diabetes.* (Female, 42 years)

### 3.5. Existential

The existential dimension describes “values and beliefs that are present in the culture and are harmless to health” [24]. The preparation of food is culturally assessed through the smell of the food—a notion that has neither negative nor positive influence in regard to diabetic risk. In this study, participants talked about the Congolese cooking methods that highlight a cultural practice to determine if foods are overcooked, undercooked, or done. Participants suggested the following:

*For me, I know that the food is ready from its smell.* (Female, 56 years)

*Since in our culture, we don't use thermometer to say that food is ready when we are cooking, I rely on smell.* (Female, 35 years)

*From its smell when I am cooking I can tell if foods are raw or done.* (Female, 42 years)

### 3.6. Negative

The negative dimension pertains to values, behaviors, and relationships that pose a negative impact on dietary habits and type 2 diabetes risk and requires immediate change in order to reduce or prevent negative health outcomes [24]. Our findings indicate that health education on type 2 diabetes is perceived as a negative factor. The paucity in exposure to and sharing of type 2 diabetes prevention knowledge can negatively influence participants' motivation to further pursue knowledge about type 2 diabetes and its preventative education. This stems from comments participants made regarding a relative lack of interest in learning more about dietary habits and type 2 diabetes risk. Participants made comments such as the following:

*No, as there is no one in our family who has diabetes. I never got interested in knowing more about the relationship that exists between diet and diabetes onset.* (Male, 47 years)

*We try to share with them (children) as I do not have someone who has diabetes, I do not really have more information on that [impact of food on diabetes risks].* (Female, 46 years)

Table 3 below summarizes and highlights the interaction that exists between the two selected PEN-3 model tenets (i.e., *relationships and expectations* and *cultural empowerment*).

## 4. Discussion

The results of this study revealed that Congolese immigrants have intrinsic, cultural ways of understanding and interpreting the interaction between dietary habits and risk for type 2 diabetes. For instance, they believed that type 2 diabetes is specifically related to the excessive consumption of sugary foods or drinks. While this is not the only practice that leads to type 2 diabetes, it is congruent with previous results [26] that report that sugar-sweetened beverages are associated with high risk for type 2 diabetes. However, this study's [26] findings were only associated with sugar-sweetened drinks and nothing else. In addition, the study revealed that Congolese immigrants obtained those beliefs regarding sweetened foods and type 2 diabetes from their parents and are in turn conveying those beliefs to their offspring. This is consistent with studies that demonstrate the impact of family members as primary sources of information on and assistance with health behaviors often associated with chronic diseases such as diabetes [27, 28]. This underlines the significant and influential roles that parents play in shaping the views and attitudes of their children.

Findings also showed a perceived awareness of unhealthy food products in supermarkets within participants' local communities; however, that perception does not prevent shopping in those establishments. This is due to a general lack of alternatives that are too distant, too costly, or do not carry Congolese indigenous food products. This aligns with Rodriguez et al.'s [29] findings which reported that food prices and the cost of various traditional products influence food access among immigrants in supermarkets. This can potentially affect any change or maintenance of healthy food options for Congolese immigrants.

Traditional diets are an integral aspect of the Congolese culture. The study findings illustrate how participants noted that the consumption of traditional Congolese meals was healthier when compared to the available dietary options in the US. Participants further suggested that traditional meals can even serve as protective from type 2 diabetes. Participants viewed their traditional diets as rich in organic products, prepared naturally without the use of synthetic products. On the other hand, American diet was perceived by participants to be high in calories and sugar, which they believed can cause type 2 diabetes. These findings are parallel to findings from a study on transnational African immigrants (from Nigeria and Ghana) who perceived their traditional diets to be healthier than the American diet because its preparation process permits them more control of what enters their body—therefore allowing more control of their health [30].

Additionally, participants reflected on their habits of consuming indigenous greens and vegetables to reduce blood sugar levels. While most study participants shared this belief, it does not have any scientific premises. This cultural belief led a group of scholars [31] to conduct an ongoing study on the antidiabetic properties of vegetables like *Fumbwa* (i.e., a vegetable cited by participants in the current study). Another study by Moise et al. [32] attempted to debunk the myth and found no association between a paucity in *Fumbwa* consumption and diabetic complications. This, however, was conducted only among diabetic people.

This study found that the majority of participants did not report a direct connection between type 2 diabetes risks and carbohydrate foods such as rice or beans. Those able to identify a connection between carbohydrate foods and type 2 diabetes risks learnt this information through either a diabetic friend or relative. The same trend was observed by Foley and BeLue [33], reporting on dietary management among type 2 diabetic patients in Senegal. They found that relatives of diabetic individuals were more likely to perceive a connection between carbohydrates and type 2 diabetes risks. In addition, participants' low diabetes-related knowledge resonates with another study [34], reporting that half of diabetic Nigerians had low knowledge of diabetes. Thus, our findings indicate that low diabetes knowledge not only is related to diabetic individuals, but also affects nondiabetic individuals.

Speculations on Congolese immigrants' tendency to show minimal concern about type 2 diabetes risks could be related to their societal and cultural backgrounds. Congolese immigrants come from a country where health screening is not part of the culture nor is it common practice due to poorly equipped medical infrastructures and the absence of effective health education programs [7]. This highlights the need to educate our population of interest on type 2 diabetes, as they exist within a group that is at high risk to develop the disease. Findings from this study provide the groundwork for interventionists to incorporate perceptions of diabetes risk factors among Congolese immigrants into a diabetes prevention program (Table 3). This particularly suggests the preference to preserve the consumption of Congolese traditional foods as opposed to “American foods,” which this population perceives as unhealthy.

While the findings of the study could be applied to most instances, there were limitations that should be acknowledged. First, its purposive sampling limits the generalizability of findings generated from the collected data. Second, not all Congolese aged 35 years or older living in Champaign were included in the study. Third, social desirability bias serves as a limitation because participants could respond to questions to appear favorable in relation to societal standards.

## 5. Conclusion

This study sheds light on perceptions of dietary habits among Congolese immigrants related to risks for type 2 diabetes. Using the PEN-3 cultural model as a guide, we identified positive, existential, and negative perceptions, along with the enablers, and nurturers associated with dietary habits and type 2 diabetes risks. Participants also acknowledged their own cultural approaches to understanding and interpreting the interaction between dietary habits and type 2 diabetes risks among Congolese immigrants that may influence their health-seeking practices. The findings underscore the importance of how cultural and sociocultural factors can influence the designing of culturally appropriate interventions aimed at addressing the risks for type 2 diabetes among Congolese immigrants in the US.

## Figures and Tables

**Table 1:** Interviewer guide.

Photo-elicitation questions
(1) What do you think about this picture? (2) How does it relate to your eating habits? (3) What does this picture mean to you?
In-depth interview questions
(1) When you think of the word “diabetes,” what first comes to mind?
*What influenced you to have these thoughts about diabetes?*
(2) Do you or any of your family members have experience with diabetes?
*What are their (i.e., family members) thoughts or experience with diabetes?*
*What factors do you think influence you or your family members to have these thoughts about diabetes?*
(3) Who do you go to when you have questions about diabetes?
(4) (For facilitator—according to CDC) Learning how to eat right is an important part of controlling diabetes. What does this mean to you?
*Why is it important to eat right given diabetes?*
(5) What do you think of grocery stores and supermarkets in the neighborhood where you live?
*Can you describe the ways in which these grocery stores/other food shopping places help people to eat right given diabetes?*
(6) What are your thoughts on the relationship between Congolese people's eating habits and diabetes?
(7) When was the last time you received food, nutrition, or eating information about Congolese diet and diabetes?
*(If information) Where did you receive this information?*
What did you think of the information you received?
*(If no information) What information on healthy eating would be helpful for you or Congolese people in the community about diabetes?*
*What are some places where displaying messages about diabetes to reach other Congolese people would be helpful?*
(8) How does being a Congolese person impact what (foods) you eat?
(9) Who influences what you eat on a daily basis?
(10) Can you share examples of “Congolese food.”
(11) Describe how you prepare Congolese food.
(12) What do you see as the strengths of Congolese foods with diabetes prevention?
(13) What do you see as the weaknesses of Congolese foods with diabetes prevention?

**Table 2:** Sociodemographic characteristics of Congolese immigrants by gender.

	Male	Female
	10 (*N*)	10 (*N*)
Age range (in years)		
Mean	43.7	44.7
Range	35–58	35–56
SD	6.63	5.46
Education level (%)		
High school	20	50
Vocational degree	—	10
Associate degree	40	10
Bachelor degree	40	30
Employment status (%)		
Unemployed	30	20
Employed	70	80
Marital status (%)		
Unmarried	—	20
Married	100	80
Residency status (%)		
Refugee	20	20
LPR	80	80

SD: standard deviation; LPR: legal permanent resident.

**Table 3:** Cultural beliefs and practices toward dietary habits and risk for type 2 diabetes in Congolese immigrants.

	Positive	Existential	Negative
Perceptions	(i) Maintaining traditional Congolese dietary of produces grown naturally.	(i) Cooking methods that rely on smell.	(i) Diabetes onset is only linked to the consumption of sweetened foods.

Enablers	(i) Making right food choices even though grocery stores do not always offer diversified healthy food choices. (ii) Relying on medical professionals to obtain information about diabetes.	(i) Local grocery stores allow access to various food products.	(i) Grocery stores present unhealthy food choices, specifically high sugar products.

Nurturers	(i) Parental influence on foods choice in regard to diabetes risk.	(i) Learning about the relationship with dietary habits and diabetes risk from diabetic friends or relatives.	(i) Knowledge about the relationship between eating habits and diabetes onset conveyed by parents is not always accurate. (ii) The lack of interest.
